# Experience on AMR Diagnosis and Treatment Following Liver Transplantation: Case Series

**DOI:** 10.1097/TXD.0000000000001598

**Published:** 2024-03-07

**Authors:** Yuanyi Mang, Yang Gao, Yan Yang, Mei Dong, Qian Yang, Hong Li, Jianghua Ran, Li Li, Jun Ma, Guoyu Chen, Bin Yang, Ying Xie, Yunsong Wu, Yingpeng Zhao, Shengning Zhang

**Affiliations:** 1 Department of Hepato-Biliary-Pancreatic Surgery and Liver Transplantation Center, The Calmette Affiliated Hospital of Kunming Medical University, The First People’s Hospital of Kunming, Clinical Medical Center for Organ Transplantation of Yunnan Province, Kunming, China.; 2 Hemodialysis Center, The Calmette Affiliated Hospital of Kunming Medical University, The First People’s Hospital of Kunming, Kunming, China.; 3 Department of Pathology, The Calmette Affiliated Hospital of Kunming Medical University, The First People’s Hospital of Kunming, Kunming, China.; 4 Department of Ultrasonic Medicine, The Calmette Affiliated Hospital of Kunming Medical University, The First People’s Hospital of Kunming, Kunming, China.; 5 Medical Imaging Center, The Calmette Affiliated Hospital of Kunming Medical University, The First People’s Hospital of Kunming, Kunming, China.

Transplant rejection presents a barrier to organ transplantation since it impacts therapeutic response and survival. Loss of graft function occurs following antibody-mediated rejection (AMR) due to involvement of donor-specific antibody (DSA).^1,2^ HLA-mismatched liver transplant recipients have long been known to suffer increased probability of graft function loss^3,4^ and HLA mismatches have been linked to DSA production. However, DSA was not considered clinically significant during the early work in this field.^5^ More recent studies have acknowledged that rejection of liver transplants is significantly increased when serum DSA levels are high and DSA concentrations have been associated with fibrosis and loss of function.^6^ The Banff Working Group on Liver Allograft Pathology clarified the diagnostic criteria for acute and chronic AMR following liver transplantation, redefining liver transplant damage of previously unknown cause or suspected humoral rejection as AMR.^7^ Preexisting DSAs are present in the host before transplantation and mediate acute T-cell–mediated rejection (TCMR) following surgery. De novo DSAs represent antibodies directed against the graft that are synthesized after transplantation and mediate rejection after a time lag.^8^ AMR accounts for 10% of DSA-positive recipients with graft dysfunction or loss of graft function.^9^ The relatively rare occurrence of AMR means that the influence of DSA on liver transplantation, risk factors for rejection, and treatment strategies have received little attention and remain unclear.^6^ Further clinical evidence is required to clarify clinical manifestations of AMR patients, histopathologic changes, genetic characteristics of chronic graft injury, and choice of examination. Four AMR cases from our hospital are presented during the current work, and diagnoses and treatments are scrutinized. The aim was to optimize diagnosis and treatment strategies to inform future AMR management.

## CASE REPORTS

### Case 1

**FIGURE 1.**
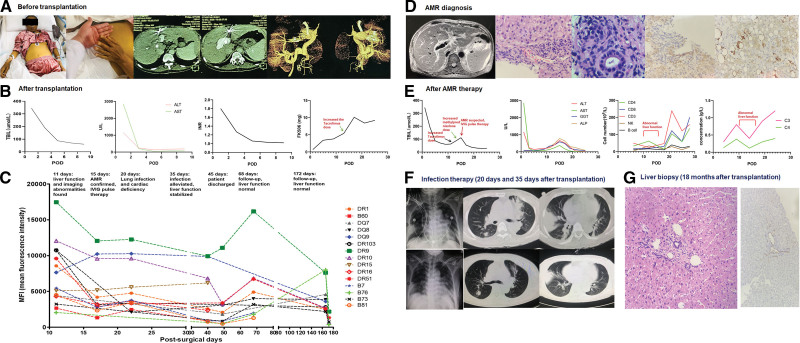
Diagnosis and therapy for a 52-y-old female patient with AMR. A, The patient’s physical condition and imaging evaluation met the indications for liver transplantation. B, All liver function indicators remained stable until the 10th day after transplantation surgery. C, The levels of all types of antibodies were high before therapy and decreased significantly following IVIG and anti-infection therapy, and remained decreasing as the liver function improved after discharge. D, Biliary duct edema was observed on MRI, and vascular endothelial swelling and multiple inflammatory cell infiltration around small blood vessels were observed by HE staining of liver biopsy. RAI score of 4, H-score of 2, and C4d score of 2 were obtained. E, Liver function, immune cell number, and C3/C4 levels continued to deteriorate after treatment with pulse methylprednisolone, tacrolimus maintenance, and mycophenolate mofetil, while they improved after switching to IVIG. F, On the 20th day after surgery, the patient developed acute pulmonary infection and acute cardiac deficiency. The infection improved and liver function was stable until the 35th day after active therapy. G, Liver biopsy was performed 18 mo after transplantation. Although C4d positive staining was still visible (score 2–3), H-score was 0, and liver function was normal. ALP, alkaline phosphatase; ALT, alanine aminotransferase; AMR, antibody-mediated rejection; AST, aspartate aminotransferase; DR, HLA-DR; DQ, HLA-DQ; GGT, gamma-glutamyl transpeptidase; HE, hematoxylin and eosin; INR, international normalized ratio; MFI, mean fluorescence intensity; NK, natural killer cell; POD, postoperative day; RAI, rejection activity index; TBIL, total bilirubin.

A 52-y-old female patient was admitted to hospital with primary biliary cirrhosis, hepatic decompensation (Child-Pugh grade C, Model for End-Stage Liver Disease [MELD] score 40), hepatic failure, hepatic encephalopathy, and esophagogastric varices. The patient’s physical condition and imaging evaluation met the indications for liver transplantation (Figure 1A). Following transplantation surgery, methylprednisolone, tacrolimus (4 mg/d, 2 mg, every 12 h [Q12h], 5.05 ng/mL), and mycophenolate mofetil were given to prevent rejection and carbapenem and third-generation cephalosporin to prevent infection. All indicators remained stable until the 10th day after surgery (Figure 1B). Bilirubin, gamma-glutamyl transpeptidase (GGT), and alkaline phosphatase (ALP) were seen to increase substantially from the 11th day postsurgery and absolute values of CD4^+^, CD8^+^, and CD3^+^ cells decreased. Viral infection and hepatotoxic drug use were excluded. Blood DSA analysis indicated 23 significantly increased antibodies (Figure 1C) and preexisting DSA was considered. MRI showed biliary duct edema and hematoxylin and eosin (HE) staining of a liver biopsy indicated vascular endothelial swelling and extensive inflammatory cell infiltration around small blood vessels (Figure 1D). Testing gave a rejection activity index (RAI) score of 4, H-score of 2, and C4d score of 2. The available evidence supported a diagnosis of AMR. Liver function continued to deteriorate after treatment with pulse methylprednisolone (500 mg), tacrolimus maintenance (2 mg, Q12h, 4.99 ng/mL), and mycophenolate mofetil (0. 5 mg, Q12h), but improved after switching to 0.5 g/kg/d IVIG (Figure 1E). The patient developed acute pulmonary infection and acute cardiac deficiency on day 20 postsurgery (Figure 1F). Immunosuppressants were discontinued for 2 d and carbapenems, linezolid, and voriconazole were given for 1 wk with IVIG at 10 g/d. Cardiotonic, diuretic, atomizing therapy, lung function exercise, and expectoration care were given. The infection improved and liver function was stable until day 35 postsurgery and the patient was discharged on day 45 (Figure 1F). Liver biopsy 18-mo posttransplantation showed C4d^+^ staining to be still visible (score 2–3), the H-score was 0, and liver function was normal (Figure 1G). High levels of all antibody types decreased significantly following IVIG and anti-infection therapy and continued to decrease as liver function improved following discharge (Figure 1C).

### Case 2

**FIGURE 2.**
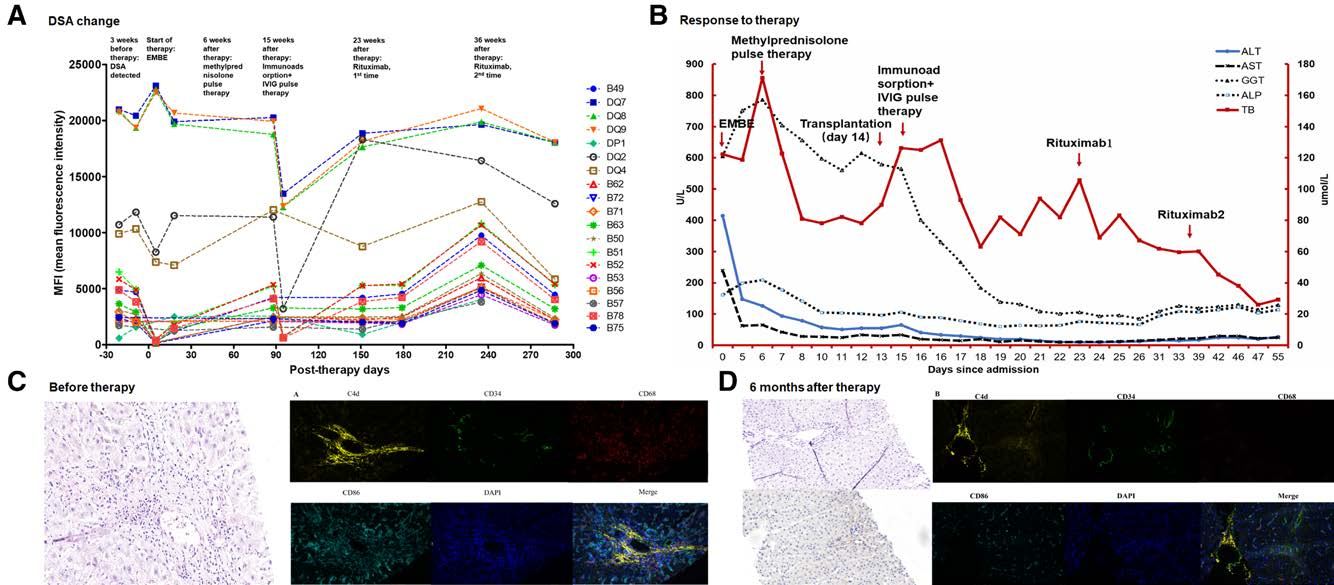
Diagnosis and therapy of a 55-y-old male patient with AMR. A, DSA changed from negative to positive in the second year after surgery, and the subtypes and MFI values continued to increase without abnormal liver function. DSA MFI levels were high before AMR therapy and fluctuated following a series of therapies, and decreased after the second time Rituximab therapy. B, Abnormal liver function was observed 4 y after transplantation. Liver function improved briefly and then continued to deteriorate after a series of therapies, while liver function was significantly relieved after CD20 monoclonal antibody was used. C, HE staining of liver biopsy showed vascular endothelial swelling, necrosis, detachment, and a large number of erythrocyte extravasation in the blood sinus and portal area with multiple inflammatory cell infiltration around small vessels. Immunofluorescence showed C4d deposition, discontinuous vascular endothelial staining, and a large number of M1 macrophages infiltration at the portal area and blood sinuses. D, After half a year of treatment, liver function returned to normal, the C4d score was 1, and the rejection was significantly relieved. ALP, alkaline phosphatase; ALT, alanine aminotransferase; AMR, antibody-mediated rejection; AST, aspartate aminotransferase; DAPI, diaminidine phenylindole; DP, HLA-DPR; DQ, HLA-DQ; DSA, donor-specific antibody; EMBE, endoscopic metal biliary endoprosthesis; GGT, gamma-glutamyl transpeptidase; HE, hematoxylin and eosin; MFI, mean fluorescence intensity; TB, total bilirubin.

A 55-y-old male patient had undergone liver transplantation 5 y previously due to hepatitis B cirrhosis and liver decompensation and recovered well after surgery. DSA changed from negative to positive in the second year after surgery and subtypes and mean fluorescence intensity (MFI) values continued to increase, although liver function was normal (Figure 2A). A long-term immunosuppressive regime of oral 5 mg/d tacrolimus (7.02 ng/mL) and 1 g/d mycophenolate mofetil were given. Progressive elevation of total bilirubin, alanine aminotransferase (ALT), aspartate aminotransferase (AST), GGT, and ALP were seen 4 y posttransplantation, indicating abnormal liver function (Figure 2B). Recurrence of viral hepatitis, cytomegalovirus infection, and vascular complications were excluded. Magnetic resonance cholangiopancreatography (MRCP) indicated an extrahepatic biliary stenosis and bilirubin continued to increase after endoscopic retrograde cholangiopancreatography imaging and biliary stent implantation. HE staining of a liver biopsy showed vascular endothelial swelling, necrosis, detachment and extensive erythrocyte extravasation in the blood sinus and portal area with inflammatory cell infiltration around small vessels (Figure 2C). The RAI score was 5–6 and H-score was 2–3. C4d was negative by immunohistochemistry (IHC) but immunofluorescence showed C4d deposition, discontinuous vascular endothelial staining, and infiltration by many M1 macrophages at the portal area and blood sinuses (Figure 2C). Administration of 500 mg methylprednisolone resulted in a brief improvement of liver function, which then resumed deterioration (Figure 2B). AMR was diagnosed. Pulse methylprednisolone therapy and immunosorbent 1 g/kg/d IVIG pulse produced a temporary improvement and administration of 375 mg/m^2^ of the CD20 monoclonal antibody, Rituximab, every 2 wk for a total of 2 doses produced a significant increase in liver function (Figure 2B). Liver function had returned to normal after 6 mo of treatment, a C4d score of 1 was seen, and rejection was significantly relieved (Figure 2D). MFI showed high DSA levels before AMR therapy and fluctuations following the series of therapies with a decrease after the second round of Rituximab therapy (Figure 2A).

### Case 3

**FIGURE 3.**
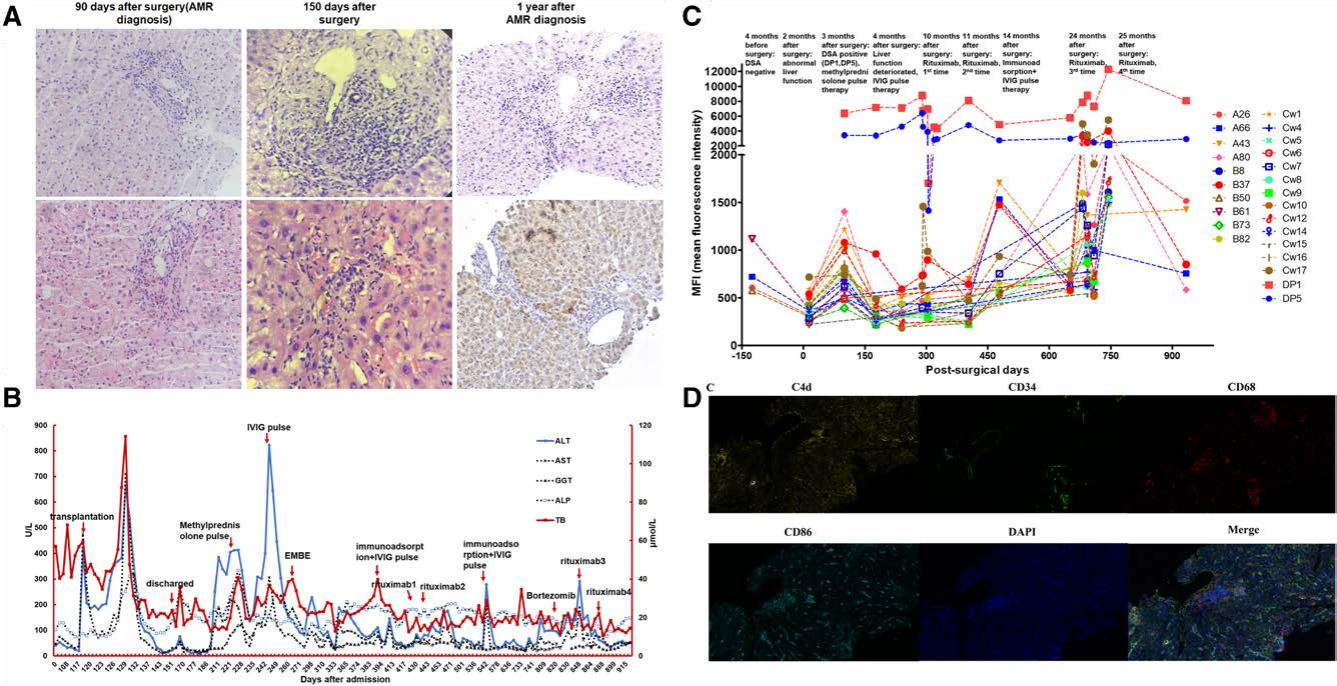
Diagnosis and therapy of a 61-y-old male patient with AMR. A, T pathological examination showed RAI score of 4, H-score of 1, and IHC C4d negative 90 d after surgery. Biopsy was performed again 150 d after surgery; the RAI score increased to 5–6, and the H-score increased to 2–3, with erythrocyte extravasation in the perisinusoidal space (Disse space) and endothelial plasma cell infiltration. The C4d score remained negative under IHC 1 y after AMR diagnosis. B, Total bilirubin, ALT, AST, and GGT elevated 90 d after surgery. Liver function returned to normal after 1 wk of treatment. Six months after the transplantation, the total bilirubin ALT, AST, GGT, and ALP elevated with sustained platelet reduction and decreased C4d. A series of therapies improved liver function temporarily. Liver function abnormalities occurred again 1 y after implantation, and returned to normal after repeated rituximab was applied. C, Progressive increase of DP1 and DP5 supported de novo DSA. DP1 and DP5 decreased to 2000 after the second rituximab administration. D, Immunofluorescence showed C4d deposition (C4 score was 2–3) and M1 macrophage infiltration. ALP, alkaline phosphatase; ALT, alanine aminotransferase; AMR, antibody-mediated rejection; AST, aspartate aminotransferase; Cw, HLA-Cw; DAPI, diaminidine phenylindole; DP1, HLA-DP1; DP5, HLA-DP5; DSA, donor-specific antibody; EMBE, endoscopic metal biliary endoprosthesis; GGT, gamma-glutamyl transpeptidase; IHC, immunohistochemistry; MFI, mean fluorescence intensity; RAI, rejection activity index; TB, total bilirubin.

A 61-y-old male patient with alcoholic cirrhosis and hepatocellular carcinoma underwent liver transplantation. A blood screen showed total bilirubin, ALT, AST, and GGT to be elevated 90 d after surgery and pathological examination gave a RAI score of 4, H-score of 1, and IHC C4d negative (Figure 3A). The observed progressive increase of HLA-DP1 (DP1) and HLA-DP5 (DP5) supported the presence of de novo DSA and AMR was considered (Figures 3B and C). Pulse 500 mg methylprednisolone, 7 mg oral tacrolimus (8 ng/mL tacrolimus), and 0.75 g mycophenolate mofetil, Q12h were given. Liver function returned to normal after 1 wk of treatment. Total bilirubin, ALT, AST, GGT, and ALP were elevated with sustained platelet reduction and decreased C4d 6-mo posttransplantation (Figure 3B). Pulse therapy with 1 g/kg/d IVIG produced a temporary improvement in liver function but repeated bilirubin and aminotransferase elevation was seen and MRCP showed extrahepatic biliary tract stenosis (Figure 3B). Immunosuppressants and long-term methylprednisolone were given and biliary stent implantation performed but liver function did not improve (Figure 3B). Biopsy 150 d postsurgery gave a RAI score of 5–6 and H-score of 2–3 with erythrocyte extravasation in the perisinusoidal (Disse) space and endothelial plasma cell infiltration (Figure 3A). Immunosorbent therapy with 1 g/kg/d IVIG produced a temporary improvement in liver function but a rebound of DP1 and DP5 was seen after 4 d of treatment (Figure 3C). Liver function was restored by the administration of 7 mg tacrolimus daily (9.39 ng/mL), 1.5 g mycophenolate mofetil daily, and 375 mg/m^2^ rituximab once every 2 wk for a total of 2 doses. DP1 and DP5 decreased to 2000 after the second rituximab administration (Figure 3C). Liver function deteriorated 1-y posttransplantation (Figure 3B) and rising levels of DP1 and DP5 were seen (Figure 3C). The C4d score remained negative by IHC testing (Figure 3A) but immunofluorescence analysis showed C4d deposition (C4 score: 2–3) and M1 macrophage infiltration (Figure 3D). Repeated doses of 375 mg/m^2^ rituximab were given and liver function returned to normal (Figure 3B). The patient’s condition remained stable afterward.

### Case 4

**FIGURE 4.**
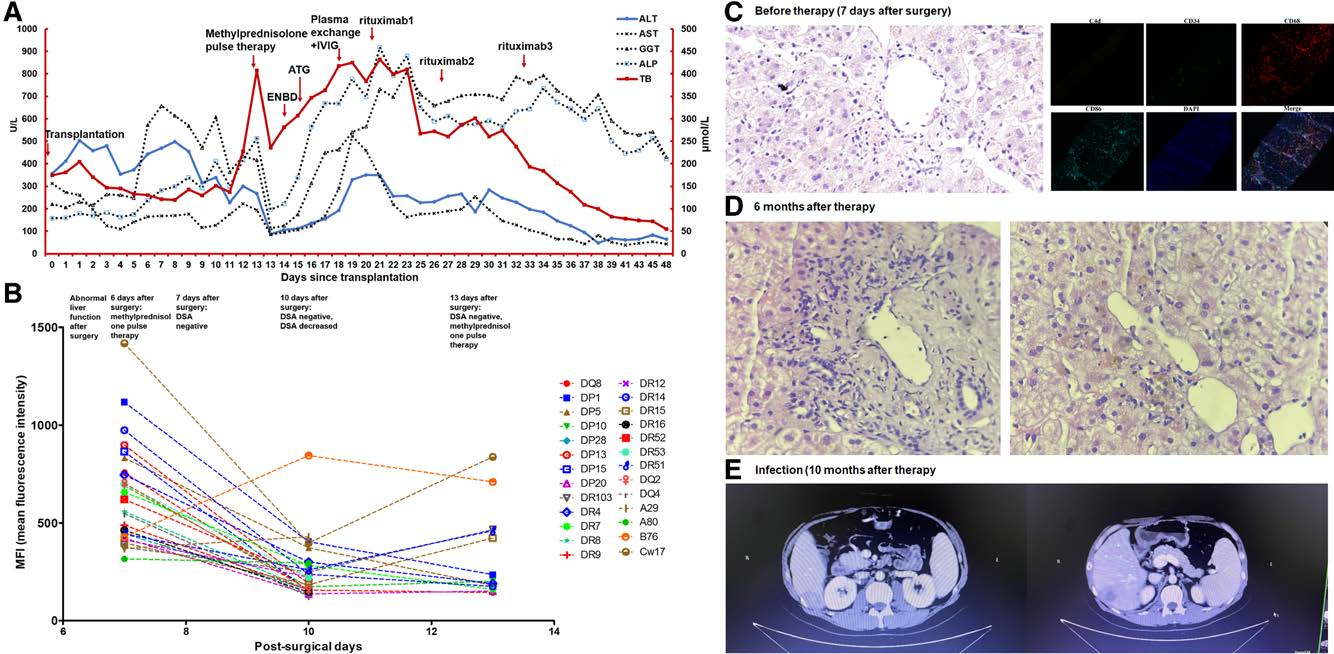
Diagnosis and therapy of a 34-y-old male patient with suspected AMR. A, Aminotransferases and bilirubin increased continuously, accompanied by fever after surgery. Liver function continued to deteriorate after a series of therapy. The patient’s liver function returned to normal after treatment with rituximab. Six months after transplantation, ALT and AST returned to the normal range, but GGT and ALP were high. B, DSA in peripheral blood remained low after surgery. C, The pathological examination showed RAI of 3, H-score of 3 with series microvascular inflammation and M1 cell infiltration. C4d was negative as assessed by IHC, and C4d score was 0 by immunofluorescence. D, The blood vessel inflammation was significantly improved, with an H-score of 1–2 after 6-mo therapy. E, The patient developed diarrhea and fever with a liver abscess after a trip and died 5 d after admission due to sepsis syndrome. ALP, alkaline phosphatase; ALT, alanine aminotransferase; AMR, antibody-mediated rejection; AST, aspartate aminotransferase; ATG, antithymocyte globulin; Cw, HLA-Cw; DAPI, diaminidine phenylindole; DP, HLA-DP; DQ, HLA-DQ, DR, HLA-DR; DSA, donor-specific antibody; ENBD, endoscopic nasobiliary drainage; IHC, immunohistochemistry; MFI, mean fluorescence intensity; RAI, rejection activity index; TB, total bilirubin.

A 34-y-old male patient with primary sclerosing cholangitis and cirrhosis underwent liver transplantation. Total bilirubin ALT, AST, GGT, and ALP showed a continuous increase and fever was present following surgery. Immunosuppressant therapy of 4 mg/d tacrolimus (5.53 ng/mL), 1.5 g/d mycophenolate mofetil, and 500 mg pulse methylprednisolone was given but liver function continued to deteriorate (Figure 4A). DSA in peripheral blood remained low after surgery (Figure 4B). An endoscopic nasobiliary drainage (ENBD) tube was inserted but no significant improvement in liver function was seen. Pathological examination showed a RAI of 3 and H-score of 3 with microvascular inflammation and M1 cell infiltration. C4d was negative as assessed by IHC and the C4d score was 0 by immunofluorescence (Figure 4C). Plasma exchange and IVIG treatment were given, followed by antithymocyte globulin treatment but the patient did not improve and total bilirubin was 400 μmol/L (Figure 4A). DSA and C4d scores were negative, but the H-score was 3, and vascular endothelial inflammation could be seen, meaning that acute AMR could not be excluded. Liver function returned to normal after treatment with a total of 3 doses of rituximab at weekly intervals (Figure 4A). ALT and AST had returned to the normal range 6-mo posttransplantation but GGT and ALP remained around 400 μmol/L. Total bilirubin was 45 μmol/L and complement and platelet levels returned to normal. Blood vessel inflammation was significantly improved with H-score of 1–2 (Figure 4D). Immunosuppressant therapy of 5 mg/d tacrolimus (5.92 ng/mL) and 1.5 g/d mycophenolate mofetil capsules was continued and B-cell levels remained undetectable 10 mo after transplantation. The patient developed diarrhea and fever with liver abscess after a trip and died 5 d after admission due to sepsis syndrome (Figure 4E). Blood metagenomic sequencing suggested adenovirus infection.

## DISCUSSION

Early identification and diagnosis of AMR is vital to a successful treatment outcome.^10-12^ Attention should be paid to indicators of early rejection, including asymptomatic liver function changes and biliary obstruction or biliary dilation, and the cause should be actively investigated by MRCP, if necessary. Number of lymph cells, concentrations of immunosuppressants, failure to take medication, and reduced absorption should be monitored and relevant indicators of opportunistic infections (cytomegalovirus/Epstein-Barr virus, etc) investigated. A prompt liver biopsy should be performed for patients with suspected AMR.^13^

A definitive diagnosis of AMR should include clear histopathologic findings, diffuse microvascular C4d, positive DSA, and exclusion of other possible causes.^7^ A positive DSA and C4d^+^ H-score of 3 or 4 would be suggestive of AMR.^7^ A C4d^+^ H-score of ≥2 with indeterminate or negative DSA or C4d may also suggest AMR, if other causes have been excluded.^7^ Suspected AMR following liver transplantation should be investigated through liver function tests and MRI evaluation. Positive imaging findings may include biliary edema and dilatation from MRCP and endoscopic retrograde cholangiopancreatography. Histological findings from HE staining of a liver biopsy may include RAI score, H-score, IHC, or immunofluorescence evaluation of C4d. Antibody testing should also be performed with preoperative and postoperative comparisons to determine the type of DSA. Not all cases of AMR gave positive C4d scores. The possibility of acute AMR should be considered even with negative C4d or DSA findings if all other causes of liver dysfunction have been excluded and histopathologic evidence of AMR exists.^7^ A definitive diagnosis was made for cases 1, 2, and 3 of the current study. Only case 1 gave positive findings of C4d by IHC and negative IHC results together with positive immunofluorescence staining were found for cases 2 and case 3. The diagnosis of AMR was indeterminate for case 4, due to negative DSA and C4d staining. However, the patient showed an H-score of 3, vascular endothelial destruction, and extensive M1 infiltration, and AMR was still suspected. Immunofluorescent detection of C4d is clearly more sensitive than by IHC.

Recent studies of DSA-related AMR following liver transplantation have focused on detailed classification of graft pathology, accurate diagnosis, DSA subtypes, quantification, binding sites, and specific functions.^14-16^ Preexisting DSA may result in TCMR of transplanted liver.^5,17^ A high MELD score, sustained use of blood products before transplantation, female gender, and autoimmune hepatitis pathogenesis are risk factors for the generation of preexisting DSA.^17^ Preexisting DSA with MFI >5000 significantly increased the 5-y mortality of transplant recipients.^18^ De novo DSA results from late-onset rejection and high levels of de novo DSA increase liver fibrosis and chronic graft function loss.^19^

HLA matching is not currently a routine procedure in liver transplantation in contrast with kidney transplants. Instead, donor liver is allocated based on Child-Pugh and MELD scores, whereas donor’s kidneys are allocated by HLA matching. Some of the current patients received plasma exchange or immunoadsorption therapy before AMR was diagnosed, and nonspecific anti-HLA antibodies may have been generated. However, antibody levels, which may have included DP1, DP5, HLA-DQ7, and HLA-DR9, fluctuated with the patient’s condition, suggesting the possibility of DSA. The current case reports suggest the potential utility of HLA matching for patients at risk of developing AMR. Testing for DSA should be considered as a routine procedure to distinguish nonspecific anti-HLA antibodies from DSA.

Glucocorticoid pulse therapy, plasma exchange plus polyclonal immunoglobulin infusion, rituximab, bortezomib, and thymoglobulin have been used to treat acute AMR in patients with high DSA levels.^20,21^ However, it is difficult to form definite conclusions as to which treatment method might have the most favorable results. The balance between immunosuppression and prevention of severe infections must be considered.^5,6^ Triple therapy with calcineurin inhibitors, such as tacrolimus, combined with mycophenolate drugs, such as mycophenolate mofetil capsules, and glucocorticoids, such as methylprednisolone, represents the current standard immunosuppressive regimen following liver transplantation.^7^ However, triple therapy does not produce a sustained effect on AMR patients in our experience and most patients showed temporary remission followed by aggravation. Triple therapy is not a specific treatment for AMR and is appropriate as the first-line treatment for TCMR in cases where AMR is undiagnosed or unclear. The temporary nature of the efficacy means that second line and multiline treatment strategies should be considered in advance.^7^ Immunoadsorption combined with IVIG pulse therapy may be considered when AMR is suspected or confirmed. Immunoadsorption is specific to AMR, resulting in the clearance of antibodies and sensitizers, and IVIG stimulates immunity and relieves hypoproteinemia and edema after liver transplantation. Plasma exchange may also be considered but is less effective than immunoadsorption and used less frequently. Immunoadsorption in combination with IVIG pulse is also suitable for repeated use or combination with other therapies, based on concentrations of antibodies and sensitizing factors in the blood. Immunoadsorption with IVIG pulse was used to stabilize liver function in case 3. CD20 monoclonal antibodies proved to be the most effective and stable treatment strategy for the current cases and repeated use of CD20 monoclonal antibodies has been shown to have additive effects.^22,23^ CD20 antibodies appeared to have a specific impact on AMR. Rejection may be alleviated with standard immunosuppressant therapy or immunoadsorption with IVIG pulse (as in case 1), meaning that CD20 antibodies may not be used as a first-line strategy but remain an option when other therapies fail. ENBD should also be considered to prevent complications due to biliary obstruction when biliary obstruction and edema are suspected. ENBD is simple and safe and has the objectives of drainage, relief of obstruction, reduction of biliary pressure, and infection prevention.^24,25^ It can be used for bile observation, bile culture, and cytological examination in the diagnosis of biliary tract diseases.

Appropriate monitoring of liver function, including bilirubin, ALT, AST, GGT, and albumin, is necessary to assess the AMR patient’s response to treatment. DSA reflects general blood antibody levels, also indicating treatment response. Liver biopsy is invasive and cannot be used repeatedly but histology, RAI score, H-score, and C4d staining give an accurate picture of the extent of liver damage.

Infection is the most common posttransplantation complication and has a significant impact on survival, especially for AMR patients. Anti-rejection and/or CD20 antibody therapies suppress T and B cells making infection harder to control. Despite these complicating factors, no anti-infection guidelines have been produced for AMR. Lung infections are considered the most common, followed by abdominal and biliary tract infections.^26^ Early postoperative blood infections are related to the long indwelling time of the deep venous catheter and are often accompanied by sudden cold and high fever. The patient’s presurgical physical condition should be monitored, hypoproteinemia corrected, blood transfusion and blood loss controlled during surgery, postsurgical duration of ventilator use regulated, and the length of the intensive care unit stay reduced. Secondly, broad-spectrum antibiotics, including carbapenems and third-generation cephalosporin, should be used prophylactically or in combination.^27^ Cytomegalovirus is the most common type of viral infection in liver transplant recipients and prophylactic therapy with intravenous ganciclovir or oral valganciclovir should be given within 3 mo of liver transplantation.^28,29^ The immunosuppressant dose should be reduced if the Epstein-Barr viral load stays high or continues to rise. Thirdly, nutritional support should be modulated. Excessive intake of energy and amino acids contributes to hepatic metabolic load slowing liver recovery after transplantation. Therefore, excessive nutrient supply should be avoided. Fourthly, respiratory support should be managed to minimize ventilator-associated pneumonia due to invasion by Gram-negative bacteria and the tracheal catheter removed as soon as possible.^30^ In the event of an infection from the donor, sensitive antibiotics, including carbapenems and the third-generation cephalosporin, should be given based on blood culture or donor liver preservation fluid culture.^31^

In this study, we shared our experience in diagnosing and treating patients with AMR following liver transplantation. We found M1 polarization, endothelial damage, and inefficient Cd4 staining by IHC in our study, and we observed sensitive Cd4 staining by immunofluorescence in terms of pathological diagnosis. We also found effective CD20 antibody treatment for AMR patients and revealed that DSA test may be an important procedure for those with high risk of AMR. However, this study also had some limitations. First, this study is a case report, and a statistically significant number of cases are required to justify the findings in this study. Secondly, 4 cases are still inadequate to establish standardized diagnosis and treatment procedures, and a systematic cohort study is required in the future.
